# Tissue-specific regulation of potassium homeostasis by high doses of cationic amino acids

**DOI:** 10.1186/s40064-016-2224-3

**Published:** 2016-05-13

**Authors:** Asunción Cremades, Jesús del Rio-Garcia, Ana Lambertos, Carlos López-Garcia, Rafael Peñafiel

**Affiliations:** Department of Pharmacology, Faculty of Medicine, University of Murcia, Murcia, Spain; Department of Biochemistry, Molecular Biology B and Immunology, School of Medicine, University of Murcia, 30100 Murcia, Spain; Biomedical Research Institute of Murcia (IMIB), Murcia, Spain

**Keywords:** Potassium, l-Arginine HCl, l-Ornithine HCl, l-Lysine HCl, l-Ornithine aspartate, Difluoromethylornithine, Cationic amino acid transporter, CAT2A

## Abstract

The administration of l-arginine hydrochloride has been used for testing pituitary secretion in humans, and as an experimental model for induction of acute pancreatitis in rats and mice. Whereas in the first case, the administration of the amino acid is associated with hiperkalemia, in the model of acute pancreatitis no data are available on possible changes in potassium homeostasis. The present study shows that the acute administration to mice of l-arginine hydrochloride or other cationic amino acids almost duplicate plasma potassium levels. This effect was associated to a marked decrease of tissue potassium in both pancreas and liver. No changes were found in other tissues. These changes cannot be ascribed to the large load of chloride ions, since similar effects were produced when l-ornithine aspartate was administered. The changes in potassium levels were dependent on the dose. The displacement of intracellular potassium from the liver and pancreas to the extracellular compartment appears to be dependent on the entry of the cationic amino acid, since the administration of an equivalent dose of alfa-difluoromethyl ornithine HCl (DFMO), a non physiological analog of l-ornithine, which is poorly taken by the tissues in comparison with the physiological cationic amino acids, did not produce any change in potassium levels in pancreas and liver. The analyses of the expression of cationic amino acid transporters (CAT) suggest that the CAT-2 transporter may be implicated in the potassium/cationic amino acid interchange in liver and pancreas. The possible physiological or pathological relevance of these findings is discussed.

## Background

Potassium is the principal intracellular cation in the body. Under normal conditions approximately 98 % of total body potassium is present in the intracellular space, while 2 % is in the extracellular fluid (Forbes and Lewis 1956; Pierson et al. 1974). It plays then an important role in maintaining the electrochemical gradient across membranes (Bia and DeFronzo 1981; Young and McDonough 2009), which is critical for the functions of many cells, particularly those of excitable tissues, such as nerve and muscle (Palmer 2015). Furthermore, potassium ions participate in regulating the secretion of many hormones, such as aldosterone (Bassett et al. 2004), insulin (Dluhy et al. 1972; Hiatt et al. 1972; DeFronzo et al. 1978), growth hormone (Dluhy et al. 1972), sex hormones and gonadotropins (Sánchez-Capelo et al. 1993; Tejada et al. 1998).

Different studies revealed that the efflux of potassium from cells to the extracellular compartment was associated with the administration of l-arginine and other cationic amino acids both in intact animals (Dickerman and Walker 1964), including humans (Merimee et al. 1965; Alberti et al. 1967; Merimee et al. 1967; Massara et al. 1979), and also in isolated rat muscle (Levinsky et al. 1962). Infusion of l-arginine hydrochloride has been used to stimulate growth hormone secretion (Knof et al. 1965; Merimee et al. 1965, 1967). Although this test may be considered nonhazardous when renal function is normal, different reports have revealed that the treatment may induce dangerous increases of serum potassium concentration in uremic and diabetic patients (Hertz and Richardson 1972; Bushinsky and Gennari 1978; Massara et al. 1979). Hyperkalemia after cationic amino acid infusion has also been observed in patients receiving infusion of l-arginine and l-lysine for renal protection, during peptide radiotherapy of neuroendocrine tumours (Rolleman et al. 2003; Barone et al. 2004; Giovacchini et al. 2011). In all these cases the tissular origin of the elevation of plasma potassium concentration was not analyzed. In addition, the administration of large doses of l-arginine to mice and rats has been used as a model to induce experimental pancreatitis (Tani et al. 1990; Takács et al. 2002; Hegyi et al. 2004; Dawra et al. 2006; Hyvönen et al. 2006; Kang et al. 2014). To our knowledge, there is no data available about a possible alteration of body potassium homeostasis produced by this treatment.

The aim of the present study was to investigate the levels of potassium in plasma and in different organs after administration of l-arginine and other cationic amino acids, at the doses used to induce acute experimental pancreatitis in mice, in order to know which tissue types drive out potassium to the extracellular compartment after administration of the cationic amino acids.

## Methods

### Chemicals

l-Arginine hydrochloride, l-lysine hydrochloride, l-ornithine hydrochloride, l-ornithine aspartate, l-glucose and primers were purchased from Sigma Aldrich. Difluoromethyl ornithine (DFMO) was kindly supplied by Dr Patrick M Woster (Wayne State University).

### Animals and treatments

Adult male Swiss CD1 mice were purchased from the University of Murcia Laboratory Animal Facilities. All the animals were housed in standard box cages in a climate controlled room with an ambient temperature of 22 ± 1 °C and 12:12 h light–dark cycle. Animals were fed standard laboratory chow, given water ad libitum and randomly assigned to control or experimental groups. All animal procedures were compliant with the national and European guidelines of animal welfare and approved by the Bioethics Committee of the University of Murcia (548/2011).

Sterile solutions of cationic amino acids (l-arginine, l-lysine, l-ornithine) and DFMO were prepared in normal saline, and the pH was adjusted to 7.0. They were administered intraperitoneally to non fasted mice at a dose of 4 g/kg. l-ornithine was also administered a doses of 2 and 3 g/kg. Animals were returned to the cages and allowed free access to food and water. After 1 h hour a second dose of the corresponding amino acid was administered. Control animals received sham injections of saline alone. Two hours after the last injection, animals were anesthetized with sodium pentobarbital and blood samples were collected by cardiac puncture. Animals were then killed by cervical dislocation, and tissues were quickly removed, weighed and processed.

### Potassium content analysis of plasma and tissues

Plasma was obtained by centrifugation of blood samples at 7000×*g* for 5 min. Tissues and plasma were homogenized in 0.3 M trichloroacetic acid (1:10 wt/vol) using a tissue homogenizer (Polytron), and the extracts were centrifuged at 7000×*g* for 10 min. Potassium content was determined using flame photometry.

### Real-time RT-PCR analysis

Total RNA was extracted from tissues using the TRIzol Plus RNA Purif Kit (Invitrogen) according to the manufacturer’s instruction. First-strand cDNA was generated from 5 μg of total RNA using (dT)18 as primer and MMLV-Reverse Transcriptase (Sigma). Real-time RT-PCR was carried out using a SYBR Green^®^ PCR Master Mix and a 7500 Real-Time instrument (Applied Biosystems). Different sets of primers and cDNA were used and the fluorescence data were collected and analyzed by means of 7500 SDS software (Applied Biosystems). The following primers were used: β-actin (forward, 5′-GATTACTGCTCTGGCTCCTAGCA-3′; reverse, 5′-GCTCAGGAGGAGCAA TGATCTT-3′); CAT1 (forward, 5′-TCACTGGCTGGAACCTGATTCT-3′; reverse, 5′-CTCTCCGATGGGCTTGCCTA-3′); CAT2A (forward, 5′-GCTCCCTCTG CGCCTTATC-3′; reverse, 5′-TCTAAACAGTAAGCCATCCCGG-3′); CAT2B (forward, 5′-TCGGCAGGCTCCCTCT-3′; reverse, 5′-CACTGCACCCG ACGACA-3′). The expression level of each gene was normalized against the housekeeping gene β-actin.

### Statistic analysis

Results are expressed as the mean ± SEM. Statically analysis was done by applying unpaired two-tailed Students *t* test with significance assigned to P values <0.05

## Results

**Fig. 1:**
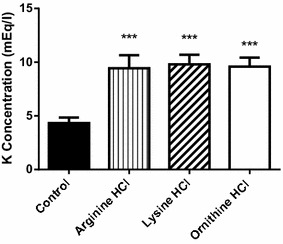
Effects of the administration of cationic amino acids to mice on plasma levels of potassium. l-Arginine HCl, l-lysine HCl and l-ornithine HCl (4 g/kg) were given intraperitoneally (two injections, 1 h apart) and plasma potassium was determined 2 h after the last injection. Results are given as mean ± SE from a minimum of six animals per group. Statistical significance: (***) P < 0.0001 versus control

The results presented in Fig. 1 show that the administration of cationic amino acids l-arginine HCl, l-lysine HCl or l-ornithine HCl (4 g/kg, i.p. twice in 1 h interval) produced a very significant rise in plasma potassium levels when measured 2 h after the last administration. The three cationic amino acids tested showed a similar increase in potassium plasma levels, reaching values close to 10 mEq/L.

**Fig. 2:**
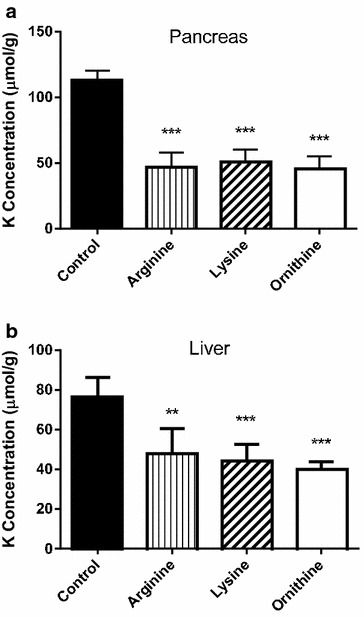
Effects of cationic amino acids administration to mice on pancreas (**a**) and liver (**b**) potassium levels. l-Arginine HCl, l-lysine HCl and l-ornithine HCl (4 g/kg) were given intraperitoneally (two injections, 1 h apart) and tissue potassium was determined 2 h after the last injection. Results are given as mean ± SE from a minimum of six animals per group. Statistical significance: (**) P < 0.001, (***) P < 0.0001 versus control

Figure 2 shows that the administration of l-arginine HCl, l-lysine HCl or l-ornithine induced a very significant decrease of potassium levels in pancreas (panel A) and liver (panel B). The decrease of the potassium content in the pancreas (55–60 %) was higher than that found in the liver (37–46 %).

Table 1 shows the potassium levels in other different tissues after the administration of the cationic amino acids l-arginine HCl, l-lysine HCl or l-ornithine HCl to mice. In contrast with the big changes found in potassium levels in plasma, pancreas and liver, the treatment only produced small potassium changes in the brain, heart, spleen, skeletal muscle and testis. In the kidney, lysine and ornithine but not arginine, produced a slight but significant decrease in potassium levels (10 % for lysine and 23 % for ornithine). Conversely, all these amino acids produced a small but significant rise in potassium brain levels (~10 %). In the heart, lysine and ornithine but not arginine elicited a small but significant increase in potassium levels (10–15 %). A smaller increase in the potassium content was also found in the testis. In spleen and skeletal muscle, no changes in potassium levels were observed.

**Table 1:** Effects of the administration to mice of cationic amino acids on potassium levels in brain, heart, spleen, skeletal muscle, kidney and testis

	Control	Arginine HCl	Lysine HCl	Ornithine HCl
Brain	95.05 ± 1.4	103.5 ± 1.3**	110.4 ± 1.9***	108.6 ± 1.5***
Heart	75.60 ± 1.6	77.50 ± 3.0	87.60 ± 2.9*	86.20 ± 3.9*
Spleen	111.8 ± 1.7	103.6 ± 2.8	105.1 ± 2.6	113.3 ± 3.0
Muscle	97.05 ± 2.2	101.1 ± 2.9	102.3 ± 3.2	99.20 ± 2.0
Kidney	70.20 ± 2.2	68.80 ± 2.2	63.20 ± 1.1*	54.60 ± 2.5*
Testis	80.20 ± 1.3	90.60 ± 3.7*	96.00 ± 2.9*	N.D.

l-Arginine HCl, l-lysine HCl and l-ornithine HCl (4 g/kg) were given intraperitoneally (two injections, 1 h apart) and tissue potassium was determined 2 h after the last injection. Results are given as mean ± SE from a minimum of six animals per group, and are expressed as µmol/g

*ND* not determined

Statistical significance: (*) P < 0.05 versus control; (**) P < 0.01 versus control; (***) P < 0.001 versus control

**Fig. 3:**
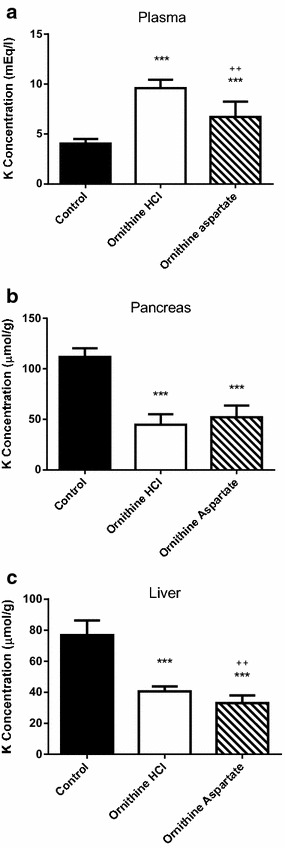
Effects of ornithine HCl and ornithine-asparte administration to mice on plasma (**a**), pancreas (**b**), and liver (**c**) potassium levels. Amino acids (4 g/kg) were given intraperitoneally (two injections, 1 h apart) and tissue potassium was determined 2 h after the last injection. Results are given as mean ± SE from a minimum of six animals. Statistical significance: (***) P < 0.0001 versus control. (^++^) P < 0.001 versus l-ornithine HCl

In order to test the possible influence of chloride ions on the potassium changes associated to the administration of the cationic amino acids under their hydrochloride forms, we compared the effect of administering equimolecular doses of either l-ornithine HCl or l-ornithine aspartate forms to mice on plasma and tissue potassium levels. As can be seen from Fig. 3, the administration of l-ornithine aspartate to mice produced marked decreases of potassium concentration in both pancreas and liver, comparable to those caused by ornithine HCl. However, as shown in Fig. 3c, the increase in plasma potassium levels after the administration of the aspartate form was significantly smaller than that found for ornithine HCl. In addition, the aspartate salt form of l-ornithine produced a significant increase in brain potassium levels (11 %) and a significant decrease of potassium levels in the kidney (23 %), with no significant changes in heart, spleen and skeletal muscle (Table 2). These effects were similar, except for the heart, to those found after the administration of an equimolecular dose of l-ornithine HCl.

**Table 2:** Effects of ornithine HCl or ornithine-asparte administration to mice on the potassium levels in brain, heart, spleen, skeletal muscle, and kidney

	Control	Ornithine HCl	Ornithine aspartate
Brain	93.14 ± 1.1	108.1 ± 1.3***	104.0 ± 1.9***
Heart	73.25 ± 1.4	84.36 ± 3.4*	78.14 ± 2.7
Spleen	112.9 ± 2.6	112.6 ± 2.0	106.0 ± 3.0
Muscle	95.14 ± 3.3	104.3 ± 3.8	104.6 ± 5.1
Kidney	69.86 ± 2.4	58.69 ± 2.0*	53.86 ± 3.2*

l-Ornithine HCl (4 g/kg) or l-ornithine aspartate (6 g/kg) were given intraperitoneally (two injections, 1 h apart) to mice and tissue potassium was determined 2 h after the last injection. Results are given as mean ± SE from six animals and are expressed as µmol/g

Statistical significance: (*) P < 0.05 versus control; (**) P < 0.001 versus control

**Fig. 4:**
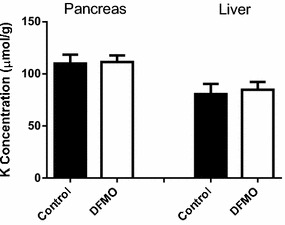
Effects of the administration of α-difluoromethylornithine HCl (DFMO) to mice on pancreas and liver potassium levels. DFMO (4 g/kg) was given intraperitoneally (two injections, 1 h apart) and tissue potassium concentration was determined 2 h after the last injection. Results are given as mean ± SE from five animals per group

To corroborate that the decrease in potassium content in pancreas and liver was associated with the uptake of the cationic amino acids, the ornithine analog alfa-difluoromethylornithine (DFMO, eflornithine), which is poorly taken up by mammalian cells most likely by passive diffusion (Grove et al. 1981; Erwin and Pegg 1982), was administered to mice (4 g/kg, i.p. twice in 1 h interval). No significant changes in potassium levels in liver and pancreas were observed (Fig. 4).

**Fig. 5:**
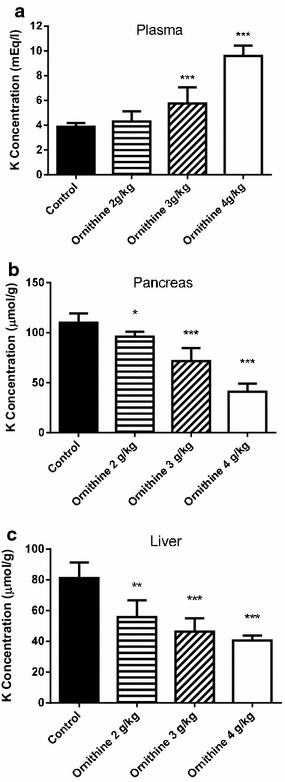
Effects of the administration of different doses of ornithine HCl to mice on plasma (**a**), pancreas (**b**) and liver (**c**) potassium levels. l-Ornithine HCl was administered at the dose of 2, 3 or 4 g/kg (two injections, 1 h apart) and tissue potassium was determined 2 h after the last injection. Results are given as mean ± SE from six animals. Statistical significance: (*) P < 0.01, (**) P < 0.001, (***) P < 0.0001 versus control

The effects of the administration of different doses of l-ornithine HCl (2, 3, and 4 g/kg, i.p. twice in 1 h interval) on plasma, liver and pancreas levels of potassium are shown in Fig. 5. l-Ornithine at the dose of 2 g/kg produced a significant decrease of potassium levels in pancreas (13 %) and in the liver (31 %), with no changes in plasma potassium concentrations (Fig. 5; panel a). However, higher doses (3 and 4 g/kg) induced a significant increased in potassium plasma concentrations accompanied with a very significant decrease in pancreas (28 % for 3 g/kg and 60 % for 4 g/kg) and liver (43 % for 3 g/kg and 50 % for 4 g/kg) potassium levels (Fig. 5).

**Fig. 6:**
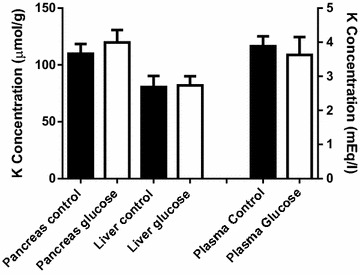
Effects of glucose administration on plasma, pancreas and liver potassium levels. d-glucose (4 g/kg) was given intraperitoneally (two injections, 1 h apart) and tissue potassium concentration was determined 2 h after the last injection. Results are given as mean ± SE from six animals per group

The determine whether the effects produced by the cationic amino acids on potassium exchange were comparable to those produced by other nutrients efficiently captured by liver and pancreas, d-glucose (at the dose of 4 g/kg i.p. twice at 1 h interval) was administered to mice. Figure 6 shows that this sugar did not produce any significant change in potassium levels, either in plasma or the tissues analyzed.

**Fig. 7:**
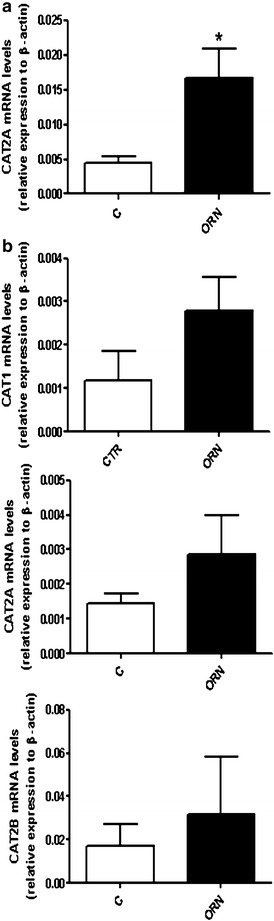
mRNA expression levels of cationic amino acid transporters (CAT1, CAT2A and CAT2B) in liver (**a**) and pancreas (**b**) of control and treated mice. l-Ornithine HCl (4 g/kg) was given intraperitoneally (two injections, 1 h apart) and tissues were excised 2 h after the last injection, and RNA was extracted and analyzed by real-time RT-PCR. Results are given as relative expression to beta-actin. Statistical significance: (*) P < 0.05 versus control of three animals per group

Since the effect of cationic amino acid administration on tissue potassium levels mainly affected liver and pancreas, the expression levels of the different cationic amino acid transporters was studied by RT-PCR in these tissues. Figure 7a shows that l-ornithine administration produced a significant increase in liver CAT-2A, the only cationic amino acid transporter detected in liver. In pancreas, the three transporters CAT 1, CAT-2A and CAT-2B were detected. In this tissue, the administration of l-ornithine showed a tendency to increase the levels of mRNA of the three transporters, although it was statistically non-significant.

## Discussion

The results of the present study indicate that the administration of high doses of cationic amino acids to mice produce a marked increase in potassium plasma levels. This is in agreement with previous observations that showed that in humans, the infusion of arginine monohydrochloride, a practice used to test pituitary function (Merimee et al. 1965, 1967), was associated with increases in plasma potassium concentration (Alberti et al. 1967; Massara et al. 1979). Similar increases have been reported in patients after the administration of l-arginine or l-lysine, used to prevent side effects induced by targeted radiolabelled peptide therapy in the treatment of neuroendocrine tumours (Barone et al. 2004; Giovacchini et al. 2011). Moreover, whereas healthy individuals developed moderate hyperkalemia upon administration of arginine HCl, severe life-threatening hyperkalemia has been reported in patients with heart disease, renal failure or diabetes (Hertz and Richardson 1972; Bushinsky and Gennari 1978; Massara et al. 1981).

Potassium is the most abundant intracellular cation in the human body and is required for vital cellular function. Serum potassium is tightly controlled through homeostatic mechanism and is affected by many factors (Young and McDonough 2009; Gumz et al. 2015). The vast majority of total body potassium is located in the intracellular space. Even small changes in the distribution between intra and extracellular compartments can result in a marked rise in serum potassium (Dickerman and Walker 1964; Evans and Greenberg 2005). Different authors have suggested that the increase in serum potassium after infusion of cationic amino acids to experimental animals or isolated tissues (Levinsky et al. 1962; Dickerman and Walker 1964) originates from the displacement of the intracellular potassium. Our results indicate that the three cationic amino acids used in the study produce similar effects on plasma potassium levels. In addition, our data clearly demonstrate, for the first time, that the increase in plasma potassium produced by the administration of the dibasic amino acids is due to the displacement of the intracellular potassium from the liver and pancreas to the extracellular compartments. This effect of the cationic amino acids on potassium levels appears to be specific of these two tissues, since no significant decreases were observed in the heart, spleen, or testes. Interestingly, the administration of the cationic amino acids did not affect potassium levels in skeletal muscle, a tissue than in other situations appears to be important for regulating plasma potassium concentrations (Gumz et al. 2015).

The unexpected increase in potassium brain levels, detected in our experiments after cationic amino acid administration, could be related with the reported effect of l-arginine administration on the release of growth hormone (GH). It is generally accepted that insufficient supply of potassium is associated with growth retardation. Thus, in potassium deficient animals both basal GH levels and GH response to GRF are reduced, this deficiency being corrected after potassium repletion (Flyvbjerg et al. 1991; Gil-Peña et al. 2010). In addition, potassium infusion to healthy man increases GH in plasma (Dluhy et al. 1972), and increased serum potassium and GH release have been observed after infusion of cationic amino acids or after physical exercises (Merimee et al. 1965; Hertz and Richardson 1972; Bushinsky and Gennari 1978; Massara et al. 1979; Williams et al. 1985; Bucci et al. 1990; Chromiak and Antonio 2002; Kanaley 2008; Denura et al. 2010).

It has been suggested that the changes in potassium homeostasis elicited by the cationic amino acids could be due to the effect of the chloride ions associated to the cationic amino acids, when they are administered under the hydrochloride form to improve solubility (Luiking and Deutz 2007). The results of the present study, showing that the administration of ornithine aspartate to mice produced similar decrease in potassium levels in pancreas and liver than when ornithine was given under the hydrochloride form, makes unlikely the possibility that chloride could be the responsible of the decrease of the potassium content in these organs. However, the smaller increase in plasma potassium concentration observed, when ornithine aspartate was administered, suggests that chloride ions may exert a negative effect on renal potassium secretion, and hence that the administration of the aspartate form of cationic amino acids could be safer than the one of chloride forms.

Our results also show that the effect of ornithine administration on potassium levels in plasma, pancreas and liver are dose dependent, suggesting that the uptake of the cationic amino acids by pancreas or liver is coupled to the exit of potassium from these tissues. The lack of effect of DFMO administration on liver or pancreas potassium levels, in contrast to the effects observed for arginine, ornithine or lysine, also support the hypothesis that the exit of potassium is necessarily associated to the uptake of the cationic amino acid, since although DFMO is a dication, as ornithine and the other dibasic amino acids, it is poorly transported into the cells by the cationic amino acid transporter (Erwin and Pegg 1982). In addition, the DFMO results also corroborate the lack of relevance of chloride ions on the potassium movements between pancreas or liver and plasma.

The capacity to drive potassium out of the cell is a property of all cationic amino acids and has been demonstrated by in vitro (Levinsky et al. 1962) and in vivo experiments (Dickerman and Walker 1964). However, the molecular bases of this phenomenon are largely unknown. Cationic amino acids are transported through biological membranes by various distinct transport systems. The main family of cationic amino acid transporters (CAT) comprises four members, CAT-1, CAT-2A, CAT-2B and CAT-3, which all exhibit a nearly identical substrate pattern for cationic amino acids (Palacin et al. 1998; Hatzogluu et al. 2004; Closs et al. 2006). CAT-1is expressed in most tissues, except in the liver (Hatzogluu et al. 2004). In contrast, the two CAT-2 splice variants exhibit a much restricted expression pattern. CAT-2A is strongly expressed in the liver and to a lesser extent, in the pancreas, skeletal muscle, heart and vascular smooth muscle cells. CAT-2A serves to remove surplus cationic amino acid content (Palacin et al. 1998; Closs et al. 2006). CAT-2B can be induced by pro-inflammatory cytokines and bacterial polysaccharides in a variety of cell lines (Palacin et al. 1998; Closs et al. 2006). Our results in mice confirm that the liver express mainly CAT-2A and that the treatment with l-ornithine produce a significant increase of hepatic CAT-2A mRNA, with no effect on the expression of the other cationic amino acid transporters. We also found a small increase of CAT-2A and CAT-2B in pancreas after l-ornithine treatment. These last findings suggest that potassium drive out from liver and pancreas could be related with the high-capacity of CAT-2A to remove surplus cationic amino acids. The molecular mechanisms by which cationic amino acid uptake is related with potassium exchange in pancreas and liver remains to be established. Different studies conducted in *Xenopus laevis* oocytes expressing hCATs showed that hCAT-2A and hCAT-2B could also transport charged particles other than cationic amino acids and that potassium can easily pass using the transporter in absence of arginine (Nawrath et al. 2000; Rotmann et al. 2004). From the results of the present study is not possible to know if after the treatment with higher doses of cationic amino acids, potassium leaks the liver and pancreas only through CATs transporters or requires an associated channel for driving potassium out of the cell.

It could be argued that the observed effects of the basic amino acids on potassium exit from liver or pancreas could be related to the formation of different products derived from the catabolism of the basic amino acids. However, whereas arginine catabolism leads to the production of several compounds, including NO, ornithine, guanidine acetate, etc., the catabolism of lysine produces different compounds (Wu et al. 2009; Wu 2013). Our results, showing that lysine and ornithine caused the same effect on potassium levels than arginine, do not support this possibility.

There are numerous animal studies in which the acute administration of large doses of l-arginine has been used as an experimental model for acute pancreatitis (Tani et al. 1990; Takács et al. 2002; Hegyi et al. 2004; Dawra et al. 2006; Hyvönen et al. 2006; Kang et al. 2014). However, potential changes in potassium homeostasis were not analyzed. There are also nutritional studies where long term dietary supplementation of l-arginine up to 3.6 g/kg body weight/day in the drinking water during 13 weeks did not show adverse effects in rats, although no data on potassium changes were available (Yang et al. 2015). In pigs, fed with supplementation of arginine (up to 630 mg l-arginine/kg/day for 91 days), potassium plasma levels did not change (Hu et al. 2015), although in this study the pancreas and liver levels of potassium were not determined. In this regard, it should be noted that in our present study we found that ornithine, at the dose of 2 g/kg body weight, induced a significant decrease of pancreas and liver potassium levels with no change in plasma levels. It is then conceivable that the alteration of potassium levels may be dependent on the rate of basic amino acid increase in plasma. In the last decades, the use of amino acids such as l-arginine, l-lysine or l-ornithine by many athletes, in order to rise growth hormone, has notably increased (Isidori et al. 1981; Bucci et al. 1990; Chromiak and Antonio 2002; Denura et al. 2010). In some cases, acute pancreatitis was observed as another hazardous effect of l-arginine supplementation (Saka et al. 2004).The possible implication of potassium changes in the pancreatic alterations observed after cationic amino acid supplementation has not been explored. However, several authors have demonstrated an association between dietary potassium and diabetes risk (Heianza et al. 2011; Chatterjee et al. 2012). The mechanism through which dietary potassium may affect glucose metabolism and diabetes risk is not well known, but several studies have evaluated that potassium depletion resulting from thiazide treatment may be the mediator of this increased risk (Zillich et al. 2006; Shafi et al. 2008). In fact, it is well documented the role of potassium for maintaining the normal secretory activity of β-cells (Bergsten et al. 1986). Furthermore, infusion of potassium either in man or in animals stimulates insulin secretion (Dluhy et al. 1972, Hiatt et al. 1972). Other factor associated with an increased risk of type2 diabetes is a high protein diet (Aune et al. 2009; Pan et al. 2011; Ericson et al. 2013). In this regard, it has been reported that the replacement of dietary protein by carbohydrates is related with a decrease in the risk of type 2 diabetes (Schulze et al. 2008). Our data reveals that the overload of glucose does not affect pancreatic potassium levels. Little is known about the reasons for the association between dietary proteins and type 2 diabetes. According to our results, one may speculate that the alteration of pancreatic potassium levels observed after administration of high amount of cationic amino acids, might result, after all, in abnormal insulin secretion. The effects of long term consumption of high protein diets on diabetes development are unknown, but the increased prevalence of type 2 diabetes in developing countries could be related with the increased intake of proteins in the diet.

In conclusion, our results demonstrate that hyperkalemia associated to high intake of cationic amino acids is related to the specific potassium depletion in liver and pancreas, and suggest that the expression of CAT-2A in these tissues may favor the interchange of potassium by cationic amino acids in these organs.
